# Supplementary material for: Patterns of Intron Gain and Loss in Fungi
Source: PLoS Biol. 2004 Nov 30;2(12):e422. doi: 10.1371/journal.pbio.0020422 (PMC532390; doi:10.1371/journal.pbio.0020422)
Supplement: Table S1 — Also available at http://genes.mit.edu/NielsenEtAl/. (4.3 MB ZIP). [file pbio.0020422.st001.zip › NielsenEtAl/html/1382.html]

AN9490.1.NCU02195.1.MG06616.1.FG00161.1


```
 CLUSTAL W (1.82) Multiple Sequence Alignments - Introns Inserted


Sequence 1: MG06616.1	610 aa
Sequence 2: FG00161.1	582 aa
Sequence 3: AN9490.1	465 aa
Sequence 4: NCU02195.1	618 aa
Alignment Length: 622 aa
Number Identitical Residues: 175 aa
Alignment Score (without introns) 11307


MG06616.1 	MAIKFWS--------RSATAADATAAADQSDSEPSVVHDGELAYTRVKAGNGSSAAYQEA
NCU02195.1	MAFNWRRPFTRRQGDDLAKSESHYRPNGPIDGDGGTVSDGSLKYTVEKGGNDSPPAYQEA
FG00161.1 	MGFSFNQ--------KSQATSQATSPGEVSESD-SIIRDGELAFVRQTAGNG-TSGYQEA
AN9490.1  	------------------------------------------------------------
          	                                                            

MG06616.1 	VGAPVESKSPLGYHVGWMTIIFLNVNQMIGTGIFSTP1ATVLNRTGSVGLALIYWLIGVI
NCU02195.1	TGAPVEVNSPLGYSVGPLSIIMLNIGKMIGTGVYSTP1SNILRGTGSVGLSMIFWALGFL
FG00161.1 	VGAPVETRSPLGYNVNWITIIFLNLNHMVGTGIFSTP~ATILKLTGSIGLALVYWVIGFI
AN9490.1  	---------------------------MVGTGIFSTP1SSVFEGTGSIGLGLFYWVIGFA
          	                           *:***::*** :.::. ***:**.:.:* :*. 

MG06616.1 	MAIAGVCIYLELASYFPNRSGGEVVYLEQAYPRPKHFFPIAFAVQSVILSFSSSNAIV~L
NCU02195.1	TSVANLSVYLEYAAYFPNRSGSEVVYLEQAYPRPRWLFPTAFAFQSVALSFSSSNAIV1L
FG00161.1 	LAVAGLAVHMEFTSYFPNRSGSEVVWLEQGYPRPKHFFPVAFAVQSVLLSFSSSNAIV~L
AN9490.1  	VAASMLSVYLEFASYFPSRSGSEAVYLEQAYPRPRYFFPTVFAVQTVVFSFSSSNAIA1-
          	 : : :.:::* ::***.***.*.*:***.****: :** .**.*:* :********.  

MG06616.1 	SRYVWRIAGTTPTEWEMRGVAIAAYTLAVVC~VVAHNKYSLWATNVIGALKIATLVF~IS
NCU02195.1	AQYLFRINGHSPTAWELKGVAVAGYTVAALF1VSFHTRLSYLFANGIGIIKVLTLIF~IS
FG00161.1 	SNYLWRIVGRAPDAWELKGVAIAAYTLAVIT~VLVHNRYSLWAINVFGALKLLLLLF~IA
AN9490.1  	-------------------------------~VSFNTKWSLQLSNALGWVKLITLIF2IA
          	                                *  :.: *    * :* :*:  *:* *:

MG06616.1 	ITGFVILGGNVGR--IPDPHRNFRNAFEG-TTTDGNSLSVALVNIVFSYTGYSNAFNMVN
NCU02195.1	ITGLVVLGGHVSTTRIPDPRINFRDAFSG--QATPYGVTNALYKIIFSYAGFENAFNVVN
FG00161.1 	ISGLVILGGNFGS--IPDPSLNFRNGFEG-TTSSGYNLSQAMVNISFAFSGWQNAFSMAN
AN9490.1  	IAGLVVLGGNVSS--ITDSTAHFRNAFEGTSNATVYGATNSLINVMFSYQGYENAFNLVN
          	*:*:*:***:..   *.*.  :**:.*.*:: :   . : :: :: *:: *:.***.:.*

MG06616.1 	EIRNPIPKLKKYATASVLIVSVLYMLCNVAYFAAVSKSEFAASKEIAAAVFFTTVFGRGG
NCU02195.1	EVKSPVKQIRRNGFVALLIVAVLYILANVAYFAAVPKDELVQSQQIAASLFFTKVFGSSG
FG00161.1 	EIKNPIPTLKKNASASLLIVFVLYFLCNIAYFAAVPKDIFLESSELAAAVFFRTAFGKS-
AN9490.1  	EVKNPVKTLKWSTPASLITVGILYMLANIAYVAAASKQEIVESEINVASLFFQKVFGGGG
          	*::.*:  ::    .::: * :**:*.*:**.**..*. :  *.  .*::** ..** ..

MG06616.1 	AETALNVLVLLSAFGNLLAVLIGSSRMIREIGR~QGVLPYTEFWVSTRPFGTPLGPYLLK
NCU02195.1	AVRGLNFLIALSSFGNLIAVLIGSSRLIRECGR2QGVLPFPRFWASTRPFGTPLGPYFVK
FG00161.1 	AESALNFCVLLSSFGNLLAVLVSQSRQIREIAR~QGVLPWTEFWVSTKPFGTPVGPYLLK
AN9490.1  	ASRALNFLICVSAFGNLLVVLIGQSRMLRECGR2QGVLPFTRFWTSTRPFGTPLGPYWLK
          	*  .**. : :*:****:.**:..** :** .* *****:..**.**:*****:*** :*

MG06616.1 	WAVTFIMIVAPPAGDAFQFV~VSLKTYPDGVFHLAMAIGVYLIRRRRKRAGMGKTEFQAW
NCU02195.1	WALTILMILAPPAGDAFNFI~TDLQVLPSAFFQLIMGIGLYVVRYRRSRLNLPRPEFKAW
FG00161.1 	WAFTFLMIVAPPAGDAFQFV~VSLKTYPDAIFFAAMGVGLLLIRRRRSRSNTPPSDFRAW
AN9490.1  	WALTAIMILAPPAGDAFNFV1VDLGIYPQSMFNFFLAVGLLFTRRHRKRLNIPRTEWHAW
          	**.* :**:********:*: ..*   *...*   :.:*: . * :*.* .   .:::**

MG06616.1 	DVVVVFFILLQVYIIAMPWWPPKGGIYAGEVSF~FYAT~YCLVGIAI2LITCGIYYYFWM
NCU02195.1	DLVVVFNILVQLYLIVMPWYPPAGGAYAGDVSF~WYGT~YVVTGIGI2LIACAIYYYLWI
FG00161.1 	HALVILYILSQIYLLVMPWIPPVGGIYGGTVSF2LWCT~YMIVGIAI~LGVCGLYYYVWM
AN9490.1  	DIVVYFAILANLYLLVAPWYPPAGGANGGDVSF~WYGT2--------~IGLCGVYYWVYM
          	. :* : ** ::*::. ** ** **  .* ***  : *          :  *.:**:.::

MG06616.1 	TLLPKWRGYRIRTEIQNVDEGSGANTHRLVRVPLANLDEWDATHDEAGFLRRRPAAAAAA
NCU02195.1	SVIPKLRGYRIRQEVLRLDD--GAQSHRLVKVPVAKLSEWDATHDAVGRPLDDSTIRPHR
FG00161.1 	KLLPRWGGYKIRSQVISVDD-NGANTHRLLKVPNDEVAEWDATHDDLG------RDLPAG
AN9490.1  	KVLPHFGGYQFRQTIITGPA--GETSHKMVKVPNDELARWDEEHDAAGR------LR---
          	.::*:  **::*  :       *  :*::::**  :: .**  **  *            

MG06616.1 	QRTASDTSSRNEGIDGVDGKDVVVPGKQAY-
NCU02195.1	GSVGSDGGSDEKKPTSADEVPLGRDGQDVEK
FG00161.1 	QRVTTEISNISK-------------------
AN9490.1  	----------RRANAHKTEQPLEA-------
          	           .
```
